# Real-time localization of percutaneous venous drainage cannula tips for cardiopulmonary bypass: A prospective trial comparing intracavitary electrocardiography and transesophageal echocardiography

**DOI:** 10.1177/11297298261468172

**Published:** 2026-09-22

**Authors:** Maria Calabrese, Mauro Pittiruti, Gabriella Arlotta, Maria Enrica Antoniucci, Temistocle Taccheri, Filippo Corsi, Natalia Pavone, Federico Cammertoni, Maria Grandinetti, Denise Derrico, Maria Giuseppina Annetta, Andrea Scapigliati

**Affiliations:** Fondazione Policlinico Universitario Agostino Gemelli IRCCS, Rome, Lazio, Italy

**Keywords:** Intracavitary electrocardiography, cardiopulmonary bypass, superior vena cava cannulation, venous drainage cannula, tip location

## Abstract

**Introduction:**

Accurate positioning of percutaneous venous drainage cannulas in the superior vena cava (SVC) is essential to ensure effective venous return during cardiopulmonary bypass (CPB), particularly in minimally invasive and re-operative cardiac surgery. Transesophageal echocardiography (TEE) is the reference standard for cannula tip localization but may not always be available or feasible. Intracavitary electrocardiography (IC-ECG) is an established technique for central venous catheter tip localization; however, its application to large-bore venous drainage cannulas used for CPB has not been previously investigated.

**Methods:**

This prospective single-center observational study enrolled 20 adult patients undergoing elective cardiac surgery requiring percutaneous SVC cannulation via the right internal jugular vein. IC-ECG signals were acquired in real-time through the cannula–introducer assembly during cannula advancement. Cannula tip position was simultaneously assessed using TEE as the reference standard. Mean P-wave amplitudes were measured at predefined anatomical locations: SVC, cavo-atrial junction (CAJ), and right atrium (RA), when feasible. Receiver operating characteristic (ROC) curve analysis was used to evaluate the diagnostic accuracy of mean P-wave amplitude in identifying correct cannula tip positioning at the CAJ, compared with SVC and RA.

**Results:**

Interpretable IC-ECG signals were obtained in all patients. No adverse events were observed. Mean P-wave amplitude was significantly higher at the CAJ compared with the SVC and RA (7.4 vs 2.6 and 5.2 mm, respectively; *p* = 0.02). ROC analysis demonstrated effective discrimination between SCV and CAJ locations with an area under the curve of 0.99 (95% CI: 0.88–1.00; *p* < 0.0001). In the group of 15 patients in whom advancement into RA was feasible, IC-ECG demonstrated high diagnostic accuracy in discriminating between CAJ and RA location.

**Conclusions:**

IC-ECG is a feasible, safe, and accurate method for real-time localization of large-bore percutaneous venous drainage cannula tips, expanding its applicability within the field of vascular access.

## Introduction

Over the past decades, advances in cardiopulmonary bypass (CPB) techniques have played a pivotal role in the evolution of cardiac surgery, particularly with the increasing adoption of minimally invasive approaches aimed at minimizing surgical trauma and improving postoperative recovery.^1^ Parallel progress in surgical and anesthetic management has extended surgical eligibility to older and higher-risk patients, resulting in a growing number of complex re-operative procedures.^2^ In these scenarios, establishing extracorporeal circulation via peripheral cannulation of arterial and venous vessels prior to sternotomy represents a critical safety strategy.^1,2^ Effective venous drainage from the upper body during CPB is commonly achieved by percutaneous cannulation of the superior vena cava (SVC) via the right internal jugular vein (IJV), using the Seldinger technique.^1–3^ Venous drainage cannulas for CPB differ substantially from conventional central venous catheters in diameter, stiffness, and distal configuration. They are advanced over a blunt, atraumatic introducer and feature a multi-holed, helical design optimized for high-volume blood drainage. In procedures requiring right atriotomy, accurate placement of the cannula tip above the cavo-atrial junction (CAJ) is essential to ensure effective SVC occlusion and adequate venous drainage.^3^ Cannula malposition may result in impaired drainage, venous congestion, increased central venous pressure, and suboptimal organ perfusion.^4^ The right IJV is the preferred access site for SVC cannulation because of its relatively consistent anatomical location, easy percutaneous access under ultrasound guidance, and alignment with the SVC, facilitating safe advancement of the cannula and reducing the risk of complications.^1,3^ Transesophageal echocardiography (TEE), particularly using the mid-esophageal bi-caval view, is currently the reference technique for confirming cannula tip position, allowing direct visualization of the terminal segment of SVC, CAJ, and right atrium (RA).^5^ Intracavitary electrocardiography (IC-ECG) is a well-known method for real-time localization of central venous catheter tips. Using the catheter as a traveling intracavitary electrode, IC-ECG detects changes in P-wave amplitude, which increases as the catheter approaches the CAJ.^6–10^ The IC-ECG method has been extensively validated for real-time catheter tip location during the insertion of centrally inserted central catheters (CICCs) and peripherally inserted central catheters (PICCs), in both adult and pediatric populations,^11,12^ and its use is supported by multiple studies, multicenter trials, international guidelines, and consensus papers^6,10,11–14^; though, its application to large-bore venous drainage cannulas has not been yet investigated. The structural features of CPB cannulas, particularly their large diameter and multi-holed distal design, may theoretically interfere with a reliable acquisition of intracavitary signals. We hypothesized that IC-ECG could be utilized for real time localization of the tip of percutaneous venous drainage cannulas by acquiring the intracavitary signal through the cannula introducer rather than through the cannula itself. The primary objective of this prospective study was to assess the accuracy of this approach in identifying correct cannula positioning at the CAJ, compared with TEE, in accordance with current recommendations on tip location.^10,13^ The secondary objective was to evaluate its technical feasibility and safety.

## Methods

### Design of the study

This prospective, single-center observational study was designed to evaluate accuracy, feasibility and safety of the IC-ECG method for real-time localization of percutaneous venous drainage cannula tips in adult patients. TEE was used as the reference standard for anatomical confirmation of cannula tip position. The primary objective was to assess the accuracy of IC-ECG in identifying correct cannula placement at the CAJ. Secondary objectives included the technical feasibility of IC-ECG and its safety. Feasibility was defined as the technical ability to obtain interpretable intracavitary P-wave signals during cannula advancement in patients with preserved sinus rhythm and P-wave morphology. Safety was assessed by monitoring for possible procedure-related adverse events, such as arrhythmias, hemodynamic instability, cannula-related complications, or other undesired events related to either TEE probe insertion or IC-ECG recording. All patients provided written informed consent prior to participation in the study, including collection and use of their clinical data for research purposes. The study protocol was reviewed and approved by the Institutional Ethics Committee (4193), and the investigation adhered to the principles of the Declaration of Helsinki and Good Clinical Practice guidelines.

### Patients

Twenty consecutive adult patients scheduled for elective cardiac surgery were prospectively enrolled. All patients required percutaneous placement of a venous drainage cannula into the SVC via the right IJV and intraoperative TEE, as part of standard clinical protocol. The inclusion criteria were as follows: (a) age >18 years; (b) written informed consent; (c) clinical indication to placement of percutaneous venous drainage cannula; and (d) planned intraoperative TEE, as recommended by the American Society of Anesthesiologists (ASA).^5^ Exclusion criteria included: (a) esophageal abnormalities contraindicating TEE probe insertion^15^; (b) pacemaker or paced rhythm; (c) atrial arrhythmias or conduction abnormalities precluding reliable P-wave analysis; (d) urgent or emergency surgery; (e) anatomical abnormalities or insufficient diameter of the right IJV preventing safe cannula placement; and (f) refusal or inability to provide written informed consent. All patients underwent standardized preoperative evaluation to confirm eligibility prior to enrollment.

### Technique

Standard intraoperative monitoring was applied upon arrival in operating room, including continuous surface electrocardiography. After induction of general anesthesia and orotracheal intubation, a TEE probe (Philips iE33 Ultrasound System with a 2.7 MHz transducer) was inserted. The mid-esophageal bi-caval view was obtained to identify the anatomical landmarks relevant for proper placement of the cannula, that is, the final tract of SVC, the CAJ (identified by the crista terminalis), and the RA. The CAJ was identified using the standard mid-esophageal bicaval view (ME bicaval view, approximately 90°–110° multiplane angle), as recommended by the ASE/SCA guidelines for comprehensive perioperative TEE.^5^ In this view, the CAJ was defined as the transition point between the tubular, thin-walled SVC and the right atrium, identified by the echogenic ridge of the crista terminalis, which appears as a distinct muscular structure at the posterolateral wall of the right atrium at the junction with the SVC. TEE imaging was continuously available during cannula advancement and served as the reference standard for anatomical localization. Percutaneous cannulation was performed via the right IJV using a modified Seldinger technique.^14^ The venous drainage device used was an Edwards OptiSite cannula, characterized by a thin-wall spiral design with multiple lateral side holes near the distal tip to optimize venous drainage during cardiopulmonary bypass. The cannula was mounted on a blunt-tip introducer, forming an integrated cannula–introducer assemblage (Figure 1). The cannula had a fixed length of 15 cm across all diameters, while the introducer extended approximately 1.5 cm beyond the cannula tip. Depth markings on the cannula allowed controlled advancement. The final position of the cannula was individualized based on patient anatomy and surgical requirements. In taller patients, advancement of the cannula tip into the RA was not always anatomically achievable due to fixed cannula length; in these cases, placement of the tip at CAJ was considered the most favorable position for venous drainage, consistent with routine clinical practice. To enable intracavitary ECG recordings, the cannula–introducer assembly was primed with 0.9% saline using a sterile Y-connector (Vygocard; Figure 2). The male Luer-lock port of the Y-connector was connected to the hub of the introducer, while the sterile cable emerging from the connector was attached to the right-shoulder electrode of the ECG monitor. This configuration allowed real-time acquisition of the IC-ECG tracing through the introducer rather than through the cannula itself. Signal acquisition via the introducer was chosen to avoid electrical signal dispersion caused by the multiple distal side holes of the venous drainage cannula and to ensure stable, high-quality P-wave recordings. The IC-ECG tracing was recorded continuously during advancement of the cannula and printed on millimeter paper at a speed of 25 mm/s. IC-ECG recordings were obtained at three predefined anatomical location, confirmed by simultaneous visualization with TEE:

**Figure 1.**
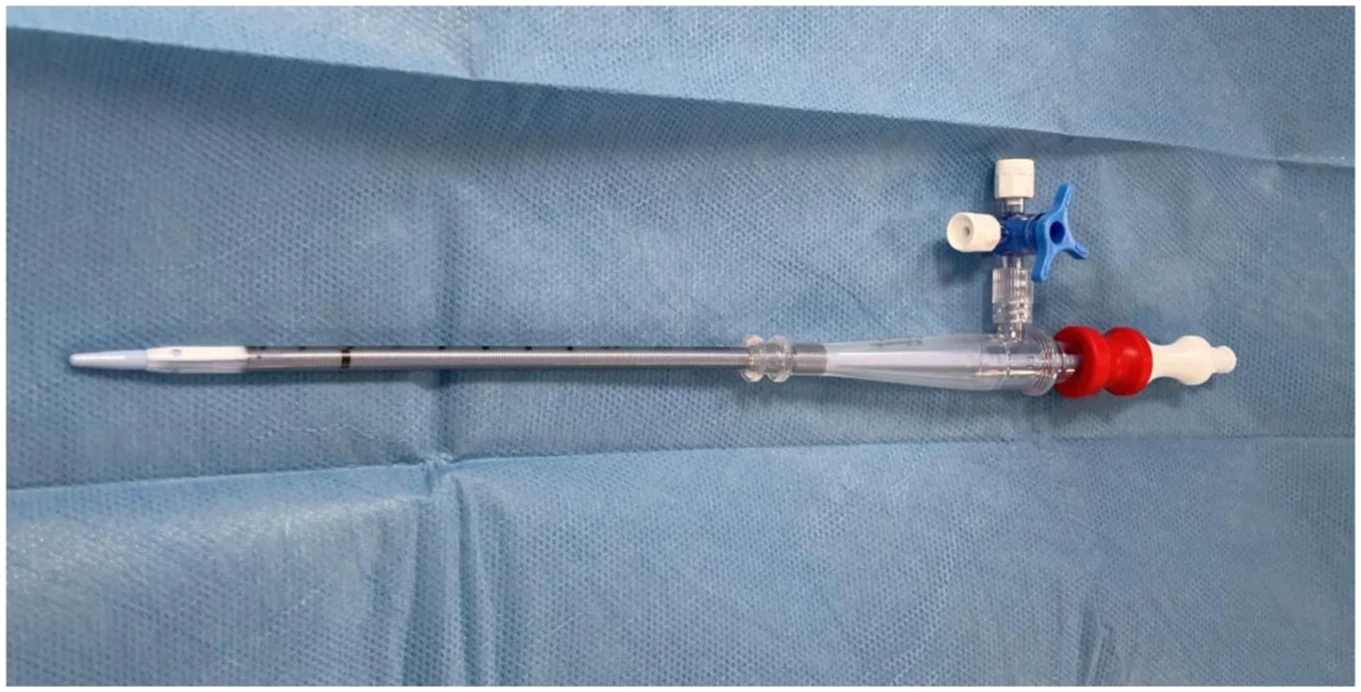
Cannula–introducer assemblage. Edwards OptiSite cannula mounted on a blunt-tip introducer, forming an integrated cannula–introducer assemblage.

**Figure 2.**
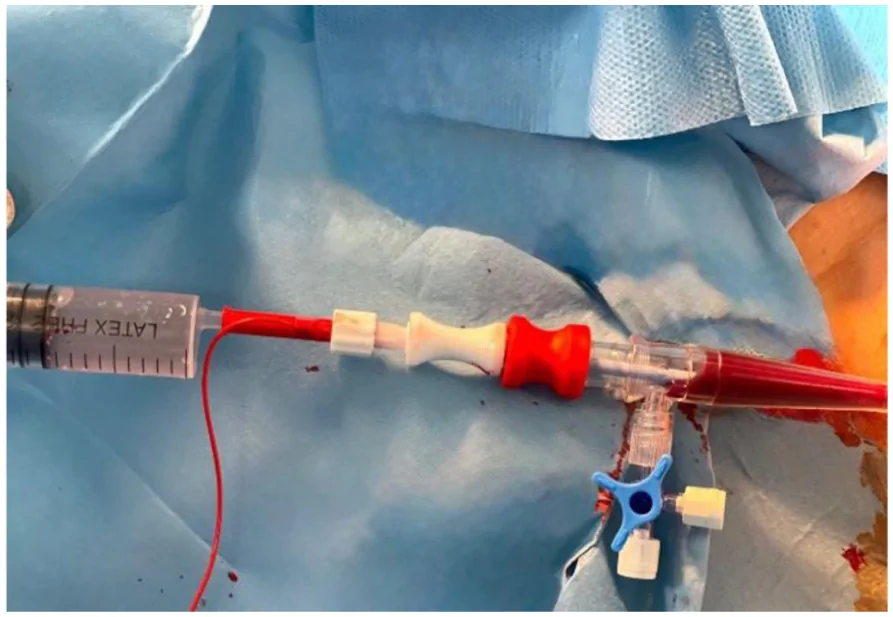
Cannula–introducer assembly.

1. approximately 2 cm proximal to the CAJ, within the superior vena cava;
2. at the CAJ, identified by the crista terminalis;
3. when anatomically reachable, approximately 2 cm distal to the CAJ within the RA.

At each anatomical position, sinus beats were recorded. P-wave amplitude was measured for each individual beat, and the values were averaged to obtain a single mean P-wave amplitude for each position in each patient. We considered the means of multiple consecutive beats for the purpose of reducing beat-to-beat variability and obtain a stable estimate of P-wave amplitude. IC-ECG tracings and TEE assessments were performed and read by two separate operators: the IC-ECG signal was acquired and interpreted by one dedicated anesthesiologist, while TEE imaging was performed and interpreted simultaneously by a second, independent anesthesiologist. Since transoesophageal echocardiography was used intra-procedurally for real-time anatomical assessment, complete operator blinding was not feasible. The anesthesiologist who performed the TEE assessment was the same for all patients included in the study. All IC-ECG traces were subsequently evaluated at the end of the study (Supplementary Material).

### Statistical and power analysis

All data are presented as mean ± standard deviation or medians with interquartile ranges, as appropriate. The Kolmogorov–Smirnov test was used to assess the normality of variable distributions. Comparisons of mean P-wave amplitude across anatomical positions were performed using one-way analysis of variance (ANOVA) on patient-level mean values, with Bonferroni correction for multiple comparisons. Receiver operating characteristic (ROC) curve analysis was used to evaluate the diagnostic performance of mean P-wave amplitude in identifying correct cannula tip positioning at the CAJ, in comparison with SVC and RA. ROC analyses were conducted using patient-level mean P-wave amplitudes. Separate analyses were performed for SVC versus CAJ location and for CAJ versus right atrial location; the latter analysis included only patients in whom right atrial advancement was anatomically feasible. The optimal P-wave amplitude cutoff was determined based on maximal diagnostic accuracy, defined as the minimization of false-positive and false-negative results. Sample size estimation was based on the primary objective of detecting an area under the ROC curve (AUC) of at least 0.76 for discrimination of CAJ positioning, with a type I error (α) of 0.05 and a power of 80%. This yielded a required sample size of 18 patients; allowing for a 10% dropout rate, 20 patients were enrolled. Two-sided *p* < 0.05 was considered statistically significant. Statistical analyses were performed using MedCalc^®^ Statistical Software (version 14.12.0; MedCalc Software bvba, Ostend, Belgium).

## Results

Twenty consecutive adult patients undergoing elective cardiac surgery and requiring percutaneous venous drainage cannulation were prospectively studied: nine males (45%) and 11 females (55%). The median age was 67 years (interquartile range (IQR): 52–72 years). The median functional class according to the New York Heart Association (NYHA) was III (IQR: 2–3; Table 1). Percutaneous venous drainage cannulation via the right IJV was successfully performed in all patients. Interpretable IC-ECG signals were obtained in 100% of cases. No patient experienced adverse events directly or indirectly related to either cannula insertion, or TEE probe placement, or IC-ECG method. Specifically, no procedure-related arrhythmias, hemodynamic instability, or cannula-related complications occurred. At each predefined anatomical position, 10 consecutive sinus beats were recorded and used to calculate the mean P wave amplitudes in each patient. Because of the fixed length of the venous drainage cannula, advancement of the cannula tip into the RA was not anatomically feasible in five patients who were taller than 180 cm: a limitation we had anticipated, being consistent with routine clinical practice. As a result, right atrial measurements were obtained only in 15 patients, whereas SVC and CAJ measurements were available in all 20 patients. This resulted in recording a total of 550 P-wave measurements: 400 measurements obtained in the SVC and at the CAJ and 150 measurements obtained in the right atrium. Mean amplitude of the P-wave differed consistently depending on the location of the tip. When the cannula-introducer was placed at the CAJ, the mean amplitude of the P-wave was significantly higher compared with measurements obtained in the SVC and RA (7.4 vs 2.6 and 5.2 mm, respectively; *p* = 0.02; Table 2). This pattern was consistent across patients and reflects the electrophysiological principles underlying IC-ECG, whereby maximal amplitude of the P-wave occurs at the CAJ. ROC curve analysis demonstrated that P-wave amplitude effectively discriminated the position of the tip among the predefined anatomical sites (Table 2 and Figure 3). ROC analysis for discrimination between SVC and CAJ, performed in all 20 patients, identified a P-wave amplitude threshold of ⩽4 mm as providing optimal diagnostic accuracy, yielding an AUC of 0.99 (95% confidence interval: 0.88–1.00; *p* < 0.0001; Figure 3). A separate ROC analysis for discrimination between CAJ and RA, performed in the 15 patients in whom right atrial advancement was anatomically feasible, similarly demonstrated high diagnostic accuracy, with narrow confidence intervals indicating robust signal separation. In patients in whom advancement of the cannula tip into the RA was not feasible, the maximal amplitude of the P-wave was consistently observed at the CAJ, as confirmed by TEE. In these cases, IC-ECG accurately identified the optimal target position for venous drainage even without entering the right atrium.

**Table 1.** Clinical characteristics of the overall study population.

Patient	Age (years)	Height (cm)	Body surface area (m^2^)	BMI	NYHA	Euroscore II (%)	Surgical procedure
1	74	165	1.7	27.5	3	1.95	Mitral and tricuspid valvuloplasty
2	43	187	2.3	26.3	3	0.96	Mitral and tricuspid valvuloplasty
3	72	175	1.8	24.5	2	0.8	MVR
4	78	160	1.3	22.7	3	1.5	MVR and tricuspid valvuloplasty
5	43	187	1.8	22.9	3	0.67	Mitral and tricuspid valvuloplasty
6	34	160	1.4		3	1.3	Tricuspid valvuloplasty
7	43	177	1.9	24.9	3	1.34	Mitral and tricuspid valvuloplasty
8	67	180	2	25	2	0.99	Mitral and tricuspid valvuloplasty
9	57	175	1.9	26.4	2	0.77	Mitral and tricuspid valvuloplasty
10	56	190	2		3	3.8	Atrial mass resection
11	84	167	1.5	23.3	2	0.7	Mitral and tricuspid valvuloplasty
12	72	170	1.8	28.7	3	1.34	MVR, tricuspid valvuloplasty
13	75	176	1.8	24.9	3	0.7	MVR, tricuspid valvuloplasty
14	68	173	1.7	25.2	3	1.73	Mitral and tricuspid valvuloplasty
15	55	180	2	24.7	3	0.67	Mitral and tricuspid valvuloplasty
16	38	165	1.3	21.3	2	1.16	Atrial mass resection
17	59	170	1.8	2	2	0.83	Mitral and tricuspid valvuloplasty
18	72	175	1.8	2	2	0.69	Mitral and tricuspid valvuloplasty
19	75	176	1.7	1.3	3	0.7	Mitral and tricuspid valvuloplasty
20	38	165	1.3	2	2	0.83	MVR

BMI: body mass index; NYHA: New York Heart Association functional class; BSA: body surface area; MVR: mitral valve replacement.

**Table 2.** Descriptive statistics of P-wave amplitude at different catheter tip positions.

Statistic	SVC (mm)	CAJ (mm)	RA (mm)^a^
Minimum	1.5	3.7	3.2
25th Percentile	2	6.2	3.8
Median	2.5	7.1	5.3
75th Percentile	3.5	8.2	6.5
Maximum	4	11.4	7.7
Means	2.6	7.4	5.2
Standard deviation	0.86	1.7	1.5
Standard error	0.21	0.43	0.44

SVC: superior vena cava; CAJ: cavo-atrial junction; RA: right atrium.

a Measurements performed in the right atrium were obtained from 15 patients.

**Figure 3.**
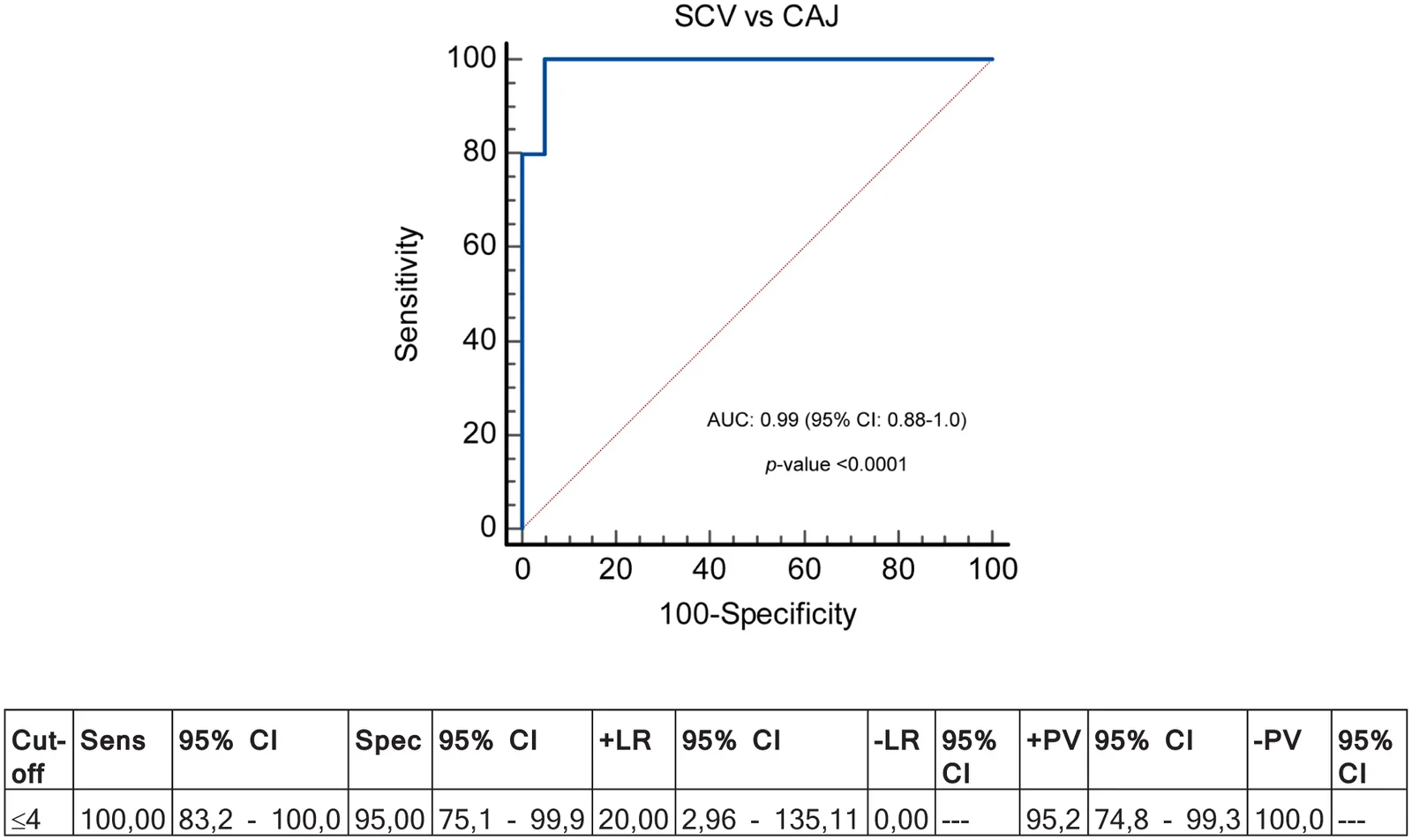
ROC curve analysis of P wave measurements for catheter tip placement in SVC versus CAJ in the overall study population. SVC: superior vena cava; CAJ: cavo-atrial junction; AUC: area under the curve; Sens: sensitivity; CI: confidence interval; Spec: specificity; +LR: positive likelihood ratio; −LR: negative likelihood ratio; +PV: positive predictive value; −PV: negative predictive value. The table below the figure presents the criterion values and coordinates of the ROC curve corresponding to the optimal cutoff identified by ROC curve analysis. +PV and −PV were calculated by accounting for the prevalence of catheter tip localization across the anatomical positions, such that the proportion of cases in the positive and negative groups reflects this prevalence.

## Discussion

This prospective observational study shows that IC-ECG can be applied for real-time localization of the tip of percutaneous venous drainage cannulas during CPB, with close concordance to anatomical confirmation by TEE. By acquiring the intracavitary signal through the cannula–introducer assemblage, the IC-ECG method can be applied to the large-bore venous drainage cannulas used for cardiopulmonary bypass. The novelty of this study lies in the extension of IC-ECG principles to large-bore venous drainage cannulas used for CPB. While IC-ECG is widely validated for CICCs and PICCs,^8,10,13^ its use in CPB cannulation has never been investigated before. Venous drainage cannulas differ substantially from standard central venous catheters in terms of diameter, stiffness, and distal geometry. They are designed for high-flow venous drainage and characterized by multiple lateral side holes near the distal tip. These structural features could theoretically impair a reliable acquisition of the electric signal. A key methodological innovation of the present study is the acquisition of IC-ECG signal through the introducer rather than through the cannula itself. This approach effectively mitigated electrical signal dispersion associated with the multi-holed cannula design and allowed the introducer to function as a traveling intracavitary electrode. As a result, stable and well-defined P-wave signals were obtained throughout cannula advancement. Regarding safety, although venous cannulation and TEE are potentially associated with procedural risks,^5^ no adverse event related to IC-ECG was observed. This finding is consistent with the extensive literature supporting the safety of IC-ECG-guided placement of central venous catheters.^8,10,13^ The absence of IC-ECG-related complications is particularly relevant in the context of complex vascular access procedures, where procedural safety is of paramount importance. The applicability of the IC-ECG method (regular sinus rhythm and normal P-wave morphology) was one of the inclusion criteria and the technical feasibility of IC-ECG was 100% in all enrolled patients. Interpretable intracavitary signals were obtained at all predefined anatomical positions. P-wave amplitude varied according to cannula tip location and showed concordance with TEE. Maximal amplitude of the P-wave was observed at the CAJ, confirming this anatomical region as the key electrophysiological landmark.^8^ Diagnostic accuracy of IC-ECG for cannula tip localization was high, as demonstrated by ROC curve analysis. For discriminating between cannula tip locations at the SVC versus CAJ, the optimal cut-off value for P-wave amplitude was identified as ⩽4 mm, yielding an AUC of 0.99 (95% CI: 0.88–1.00, *p* < 0.001). This AUC value, coupled with narrow confidence intervals, indicates that IC-ECG provides reliable and accurate discrimination of anatomical position when used through the cannula-introducer assemblage. Our analysis focused on identification of the CAJ rather than RA. This distinction is clinically relevant in taller patients, in whom advancement of a fixed-length cannula into the RA may not be anatomically feasible. In such cases, IC-ECG reliably identified the CAJ as the endpoint. While TEE remains the reference technique for anatomical visualization,^10^ its use requires specific expertise, may be contraindicated in some patients, and adds procedural complexity.^15^ IC-ECG, in contrast, offers a simple, inexpensive, and reproducible alternative that provides immediate feedback on cannula tip position without requiring complex image acquisition or manipulation of the probe. In this clinical setting, IC-ECG should be viewed as a complementary or alternative tool rather than a replacement for echocardiography. The high concordance observed between IC-ECG and TEE supports its role as a practical adjunct, particularly in settings where TEE availability is limited. Overall, this study demonstrates that IC-ECG, when appropriately performed, provides a reliable and reproducible method for real-time guidance of venous drainage cannula positioning during cardiopulmonary bypass.

### Limitations of the study

This study, while providing valuable insights into the application of IC-ECG for percutaneous venous drainage cannula tip localization, is subject to certain limitations. First, this was a single-center, prospective observational study. Second, all procedures were performed by experienced cardiac anesthesiologists in a high-volume cardiac surgery center. Future multicenter studies are warranted to confirm these findings and assess reproducibility across different operators and setting. Third, feasibility of the IC-ECG technique is inherently limited to patients with preserved P-wave morphology. Patients with atrial fibrillation, atrial flutter, paced rhythms, or significant conduction abnormalities were excluded, as reliable P-wave analysis is not possible in these conditions and alternative solutions should be investigated. Consequently, the applicability of IC-ECG cannot be extended to all cardiac surgical patients. Fourth, this study evaluated a single type of venous drainage cannula and introducer system. Cannula design, length, material, and distal geometry may influence signal acquisition and generalizability of the results. Future investigations should assess whether similar performance can be achieved with different cannula designs and manufacturers.

## Conclusions

This study supports the use of IC-ECG as a feasible, safe, and accurate method for real-time localization of large bore percutaneous venous drainage cannulas, expanding its applicability within the field of vascular access.

## Supplemental Material

sj-docx-1-jva-10.1177_11297298261468172 – Supplemental material for Real-time localization of percutaneous venous drainage cannula tips for cardiopulmonary bypass: A prospective trial comparing intracavitary electrocardiography and transesophageal echocardiography Supplemental material, sj-docx-1-jva-10.1177_11297298261468172 for Real-time localization of percutaneous venous drainage cannula tips for cardiopulmonary bypass: A prospective trial comparing intracavitary electrocardiography and transesophageal echocardiography by Maria Calabrese, Mauro Pittiruti, Gabriella Arlotta, Maria Enrica Antoniucci, Temistocle Taccheri, Filippo Corsi, Natalia Pavone, Federico Cammertoni, Maria Grandinetti, Denise Derrico, Maria Giuseppina Annetta and Andrea Scapigliati in The Journal of Vascular Access
