# Supplementary material for: Real-time localization of percutaneous venous drainage cannula tips for cardiopulmonary bypass: A prospective trial comparing intracavitary electrocardiography and transesophageal echocardiography
Source: J Vasc Access. 2026 Sep 22;27(6):2892–9. doi: 10.1177/11297298261468172 (PMC13691219; doi:10.1177/11297298261468172)
Supplement: Supplementary file 1 [file sj-docx-1-jva-10.1177_11297298261468172.docx]

**Figure Supplementary material**

IC-ECG tracings
